# Interdigital Tinea Nigra

**DOI:** 10.7759/cureus.7579

**Published:** 2020-04-07

**Authors:** Marina K Ibraheim, Michelle A McNally, Jaime Tschen

**Affiliations:** 1 Dermatology, John P. and Kathrine G. McGovern School of Medicine, University of Texas Health Center, Houston, USA; 2 Dermatology, St. Joseph Dermatopathology, Houston, USA

**Keywords:** tinea nigra, interdigital, dermatomycosis, koh preparation

## Abstract

Tinea nigra is an uncommon superficial dermatomycosis precipitated by *Hortaea werneckii*, a halophilic and halothermic yeast-like fungus capable of producing a melanin-like substance. This pathogen infiltrates the stratum corneum in the setting of microtrauma and produces an asymptomatic brown to black macule or patch that appears similarly to melanocytic nevi or melanoma. We present a case of a 52-year-old woman who presented to clinic several months after developing a painless, nonpruritic dark brown patch in her left foot inside the fourth toe web. The coloration and location of this lesion would typically prompt biopsy; however, Wood’s lamp examination and potassium hydroxide (KOH) preparation were pursued first and demonstrated evidence of infection by *H. werneckii*. The patient was treated with topical clotrimazole cream and miconazole powder for one month, and her lesions cleared completely. Her lesions did not recur at her three-month follow-up appointment.

## Introduction

Tinea nigra is a superficial dermatomycosis precipitated by *Hortaea werneckii*, a melanized yeast-like fungus formerly classified in the literature as *Exophiala*, *Phaeoannellomyces*, and *Cladosporium* [1]. This pathogen thrives in high-temperature, low-oxygen, and humid environments, affecting individuals in temperate or subtropical climates [1,2]. For this reason, palmoplantar hyperhidrosis significantly predisposes to the development of tinea nigra [1].

Patients present with asymptomatic, unilateral, enlarging, well-demarcated, brown to black macules with irregular borders on the palms or soles [1]. The lesions appear grossly similar to melanocytic lesions [3]. Utilization of KOH preparation, Wood’s lamp, and dermoscopy can distinguish between these two diseases [4]. We present a woman with interdigital tinea nigra without a prior history of melanoma; KOH testing prevented unnecessary biopsy.

## Case presentation

A 52-year-old woman presented several months after developing a painless, nonpruritic dark brown patch in her left foot inside the fourth toe web. She had never developed a lesion of this nature before. The patient’s past medical history was significant for lentigos, angiomas, and basal cell carcinoma on the nose treated two years earlier. No family members or close contacts had developed these lesions.

**Figure 1:**
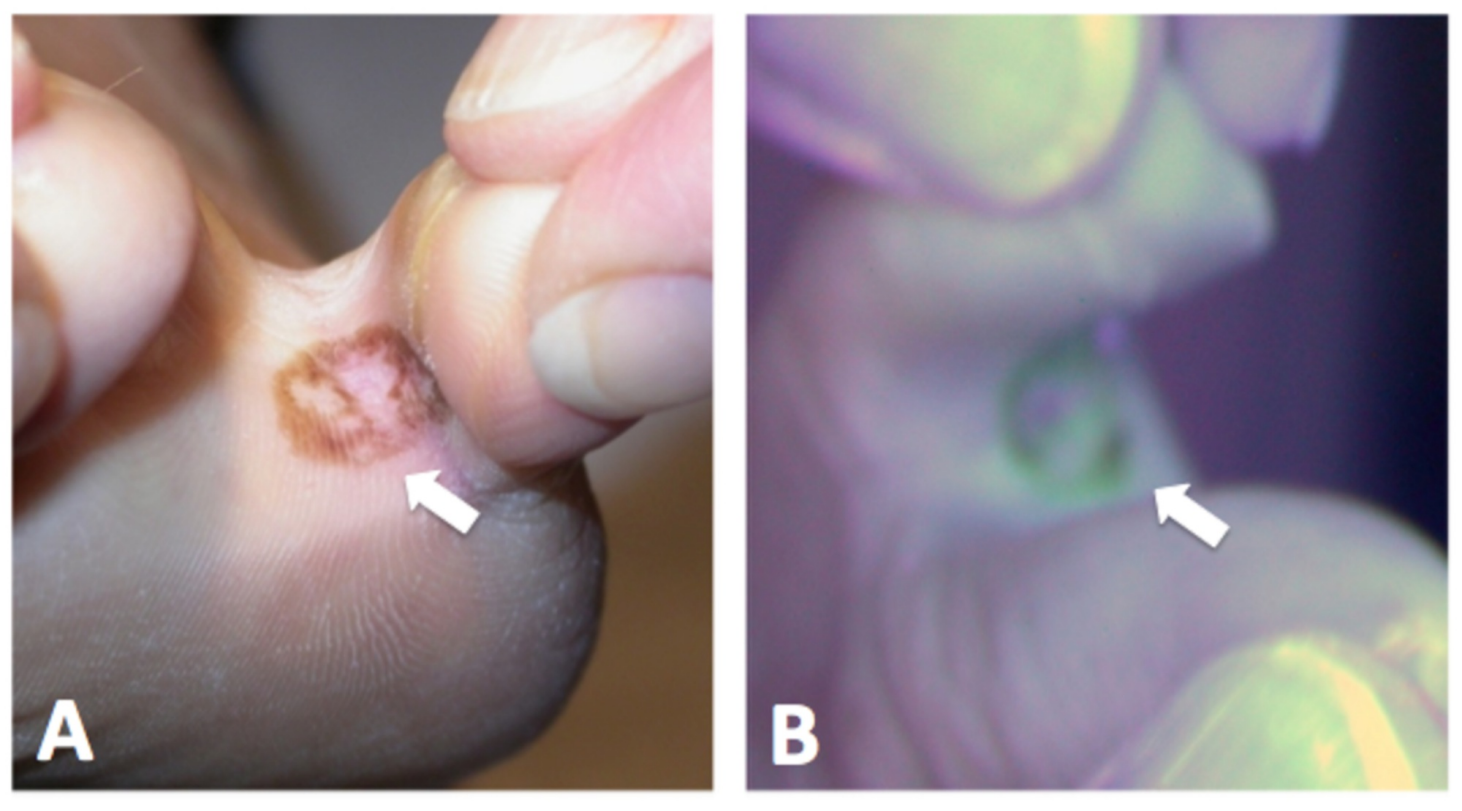
Irregular pigmented patch visualized grossly and under Wood's light (A) Brown irregular patch in the left fourth toe web. (B) Lesion fluorescing green under Wood’s light.

Physical exam revealed a brown irregular patch in her left fourth toe web (Figure 1A). Wood’s light examination showed a green fluorescence over the area (Figure 1B).

**Figure 2:**
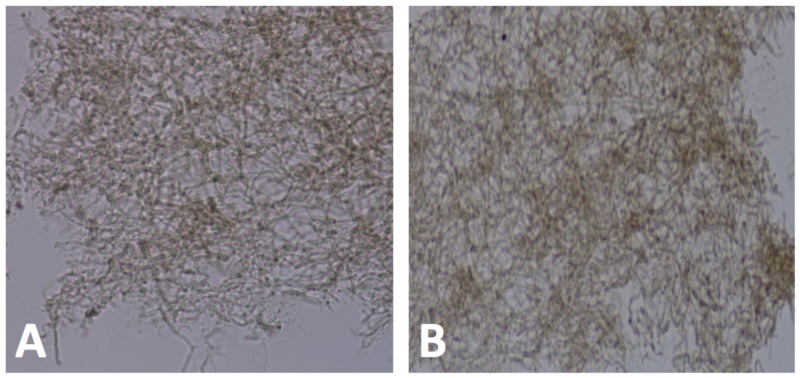
KOH scraping of lesion (A) From KOH preparation, we can see colonies of brown, septating, branching hyphae with spores. (B) Closer view.

A KOH examination of scrapings showed colonies of brown fungi both in spores and hyphae (Figure 2). 

The patient was instructed to utilize clotrimazole cream and miconazole powder for one month. After one-month follow-up, her lesion resolved; after three-month follow-up, her lesions did not recur.

## Discussion

Tinea nigra develops when *H. werneckii* inoculates the stratum corneum in the setting of microtrauma and incubates for two to seven weeks. The fungus thrives in the humidity and has affected individuals living in tropical and subtropical areas, such as Mexico, Venezuela, Chile, Brazil, India, Japan, and Panama [1,5-9]. Cases reported in the United States occur infrequently. Cases have been reported in North Carolina, South Carolina, Texas, and Florida [9-11]. Texas and Florida in particular harbor more humid environments, which enable fungal growth. 

Once the fungus has infiltrated the lipid-rich, warm, and humid environment of the stratum corneum, *H. werneckii* proliferates and develops a melanin-like substance within itself [12]. The pigmented lesion seen with tinea nigra often appears similar to melanocytic nevi or melanoma [3,12]. The patient's recent history of skin cancer prompted concern for the presence of another malignancy. The location of the patient’s lesion in this case further complicates matters, as tinea nigra classically appears on palmoplantar surfaces. Interdigital infiltration occurs infrequently [1]. Given this uncommon presentation, pigmented macular lesions like this may warrant a biopsy; however, biopsies are invasive, distressing, and results take time to return. Utilization of noninvasive modalities of testing such as dermoscopy and KOH preparation can aid in prompt diagnosis. Dermoscopic examination would show wispy brown strands, or pigmented spicules that do not follow dermatoglyphic furrows and ridges [13]. KOH preparation would reveal brown, septate hyphae, consistent with what was seen in this case [3].

Topical keratinolytic and antifungal agents are typically utilized to treat tinea nigra; hence, there is no need for systemic therapy. Remedies include undecylenic acid, Whitfield’s ointment, retinoic acid, ketoconazole, itraconazole, clotrimazole, or miconazole [12]. In our case, the combination of clotrimazole cream and miconazole powder was cost-effective and efficacious in Houston's humid climate. The infection resolves within two weeks to a month after treatment and typically does not recur [1,3,12].

## Conclusions

Tinea nigra is an uncommon dermatomycosis typically presenting on palmoplantar surfaces of the body and rarely occurs on interdigital surfaces. *H. werneckii*, the underlying fungus, develops a melanin-like substance in the stratum corneum, resulting in the development of asymptomatic brown macules and patches with irregular borders. These pigmented lesions are concerning for melanocytic nevi or melanoma. This case highlights the concern and importance in differentiating *H. werneckii* from melanoma, especially in patients with a past history of skin cancer. While biopsy can aid in diagnosis, Wood’s lamp and KOH preparation can allow for efficient and noninvasive assessment of a pigmented lesion.
